# Presence of tobramycin in blood and urine during selective decontamination of the digestive tract in critically ill patients, a prospective cohort study

**DOI:** 10.1186/cc10489

**Published:** 2011-10-17

**Authors:** Heleen M Oudemans-van Straaten, Henrik Endeman, Robert J Bosman, Milly E Attema-de Jonge, Marc L van Ogtrop, Durk F Zandstra, Eric JF Franssen

**Affiliations:** 1 Department of Intensive Care Medicine, Onze Lieve Vrouwe Gasthuis, Oosterpark 9, 1091 AC Amsterdam, The Netherlands; 2 Department of Pharmacy, Onze Lieve Vrouwe Gasthuis, Oosterpark 9, 1091 AC Amsterdam, The Netherlands; 3 Department of Clinical Microbiology, Onze Lieve Vrouwe Gasthuis, Oosterpark 9, 1091 AC Amsterdam, The Netherlands

## Abstract

**Introduction:**

Tobramycin is one of the components used for selective decontamination of the digestive tract (SDD), applied to prevent colonization and subsequent infections in critically ill patients. Tobramycin is administered in the oropharynx and gastrointestinal tract and is normally not absorbed. However, critical illness may convey gut barrier failure. The aim of the study was to assess the prevalence and amount of tobramycin leakage from the gut into the blood, to quantify tobramycin excretion in urine, and to determine the association of tobramycin leakage with markers of circulation, kidney function and other organ failure.

**Methods:**

This was a prospective observational cohort study. The setting was the 20-bed closed format-mixed ICU of a teaching hospital. The study population was critically ill patients with an expected stay of more than two days, receiving SDD with tobramycin, polymyxin-E and amphotericin-B four times daily in the oropharynx and stomach. Tobramycin concentration was measured in serum (sensitive high performance liquid chromatography - mass spectrometry/mass spectrometry (HLPC-MS/MS) assay) and 24-hour urine (conventional immunoassay), in 34 patients, 24 hours after ICU admission, and in 71 patients, once daily for 7 days. Tobramycin leakage was defined as tobramycin detected in serum at least once (> 0.05 mg/L). Ototoxicity was not monitored.

**Results:**

Of the 100 patients with available blood samples, 83 had tobramycin leakage. Median highest serum concentration for each patient was 0.12 mg/L; 99% of the patients had at least one positive urinary sample (> 0.5 mg/L), 49% had a urinary concentration ≥ 1 mg/L. The highest tobramycin serum concentration was significantly associated with vasopressor support, renal and hepatic dysfunction, and C-reactive protein. At binary logistic regression analysis, high dopamine dose and low urinary output on Day 1 were the significant predictors of tobramycin leakage. Nephrotoxicity could not be shown.

**Conclusions:**

The majority of acute critically ill patients treated with enteral tobramycin as a component of SDD had traces of tobramycin in the blood, especially those with severe shock, inflammation and subsequent acute kidney injury, suggesting loss of gut barrier and decreased renal removal. Unexpectedly, urinary tobramycin was above the therapeutic trough level in half of the patients. Nephrotoxicity could not be demonstrated.

## Introduction

Selective decontamination of the digestive tract (SDD) reduces the number of Gram-negative and yeast infections in intensive care patients and reduces hospital mortality [1,2]. It does so by eradicating Gram-negative bacteria and yeasts from the intestinal tract, while preserving Gram-positive and anaerobic bacteria. Treatment consists of the local application of tobramycin, polymyxin and amphotericin in the oropharynx and gastrointestinal tract. If the gut barrier is intact, the antibiotics exert their effect in the digestive tract and are not absorbed. However, conditions such as sepsis, shock or major surgery can convey gut barrier failure [3] and may result in the leakage of the antibiotics from the gut into the blood. Among these, tobramycin will most readily leak, since its molecular weight is the smallest of the active constituents of SDD (1,425 D). Furthermore, critical illness may additionally be associated with acute kidney injury (AKI), hampering removal of the leaked tobramycin [4]. If tobramycin leakage from the gut persists over time and attains significant concentrations in the blood, accumulation might cause harm to the kidney and the inner ear [5,6]. We hypothesized that during critical illness some of the enteral administered tobramycin leaks to the systemic circulation and is subsequently excreted in urine.

The aim of this prospective observational cohort study in critically ill patients receiving SDD was to determine whether and to what extent the enterally administered tobramycin leaks to the circulation and is excreted in urine, and whether we can predict the leakage of tobramycin with markers of circulation, kidney function and other organ failure.

## Materials and methods

### Study design and setting

This prospective observational cohort study was conducted in a 20-bed closed format general ICU of a teaching hospital. SDD is routinely prescribed to all critically ill patients with an expected ICU stay of more than two days. We first measured the presence of tobramycin in blood and urine in a cohort of 20 patients during the first day of ICU admission. Because of the great proportion of patients with tobramycin leakage and because we wanted to assess whether tobramycin leakage continued after Day 1, we continued measuring tobramycin in blood and urine in a second cohort. In this cohort, tobramycin was measured daily during the first week of ICU admission. The institutional review board approved the study according to European and Dutch legislation. Written informed consent was obtained from the patient or his legal representative *a priori *or delayed but within the first 24 hours.

### Patients

Subsequent adult critically ill patients acutely admitted to the ICU were eligible for inclusion if the expected duration of treatment with SDD was more than two days. Exclusion criteria were treatment with tobramycin or gentamicin intravenously in the 72-h preceding ICU admission and during the study period. Patients with a positive baseline tobramycin concentration were *a priori *excluded from analysis.

### Selective decontamination of the digestive tract

The SDD regimen consisted of an enteral and an oral part. At six-hour intervals a solution containing polymyxin-E 100 mg, tobramycin 80 mg and amphotericin-B 500 mg was administered via the gastric tube. At the same time, an oral paste, containing 2% polymyxin E, 2% tobramycin and 2% amphotericin B, was applied in the mouth.

### Study protocol

After inclusion in the study, a baseline blood sample was taken directly after ICU admission for measurement of tobramycin concentration (Day 1). Baseline markers of organ function were determined, and oral and rectal surveillance cultures were taken according to routine clinical practice. Thereafter, the first dose of SDD was administered via the gastric tube, and repeated at six-hour intervals until discharge from the unit. Subsequent blood samples and a portion of the 24-h urine were taken for determination of tobramycin 24-h after admission in the first cohort, and once daily for seven days in the second cohort.

### Measurement of tobramycin in serum and urine

Tobramycin in serum was quantified with a previously described and validated extremely sensitive HLPC-MS/MS assay [7]. This simple, rapid and sensitive method detects tobramycin with a lower limit of quantification of 0.05 mg/l. The accuracy was good; within-day and between-day precisions were less than 12%. For the determination of tobramycin in urine we used a conventional immunoassay method (lower limit of quantitation 0.5 mg/l) (Emit^® ^2000, Siemens Healthcare Diagnostics Inc., Newark, DE 19714, USA). Notably, the method used in serum was 10-fold more sensitive than the conventional assay used for urine.

### Clinical measurements

Apart from routine clinical measurements of circulation, gas exchange, fluid balance, renal and hepatic function, severity of illness was scored using the Acute Physiology and Chronic Health Evaluation (APACHE II, III and IV) systems [8,9] over the first 24-hours of ICU admission. The Sequential Organ Failure Assessment (SOFA) score [10] as defined by the Dutch National Intensive Care Evaluation [11] was measured daily. Renal function was classified according to the RIFLE (Risk, Injury, Failure) System [12]. Risk was scored as 1, Injury as 2 and Failure as 3.

### Endpoints and definitions

The primary endpoint of the study was the proportion of patients with tobramycin leakage into the blood circulation. Tobramycin leakage was defined as at least once a detectable serum tobramycin sample (> 0.050 mg/L), and no tobramycin leakage as no tobramycin detectible in serum during the entire study period. Secondary endpoints were the concentration of tobramycin in serum and urine, and the relation between tobramycin leakage and markers of circulation, renal and other organ function. A detectable urine concentration was defined as ≥ 0.50 mg/L. Due to the less sensitive method used in urine, the detectable concentration in urine was higher than in serum. A therapeutic trough concentration was defined as ≥ 1 mg/L [13].

### Data analysis

We analyzed the two cohorts both separately and together. When analyzing them together, the results from the second cohort became statistically stronger due to the greater sample size. Therefore, we present the data of the two cohorts together. Values are presented as mean (standard deviation, SD) and/or median (interquartile range, IQR). To compare tobramycin concentrations at different time points, we used the Wilcoxon Signed Ranks test. Due to differences in ICU stay, follow-up time varied between patients. We, therefore, analyzed two single markers of tobramycin leakage per patient. The quantitative marker, the highest serum tobramycin concentration, was used for correlation with clinical markers of circulation and organ failure at the same day and with markers on ICU admission day. The qualitative marker, tobramycin leakage (see definitions), was used to compare patients with tobramycin leakage to those without tobramycin leakage. For correlations we used the two-sided Spearman Rank test and for comparisons between groups the unpaired T-test or Mann-Whitney U test for continuous values and the two-sided Fisher's exact test for non-parametric variables.

To determine the variables independently predicting tobramycin leakage, binary logistic regression analysis was used. All of the covariates that were different at unadjusted analysis with a *P-*value < 0.10 were entered simultaneously into the model. Backward stepwise regression analysis was applied by removing the least significant value step by step until only significant determinants remained. Odds ratios (OR) and their 95% confidence intervals (95% CI) are reported. We used SPSS^® ^version 18.0 (IBM corporation, New York, USA).

## Results

A total of 105 patients was included in this study. Baseline characteristics are presented in Table 1. None of the patients received additional tobramycin suppositories or tobramycin nebulization, and in none of the patients, tobramycin dose differed from the protocol. In 34 consecutive patients (that is, 20 in the first cohort and 14 in the second), tobramycin was measured during the day of ICU admission only, and in 71 consecutive patients, tobramycin was measured daily from Day 1 to Day 7, or until discharge from the ICU. A blood sample was missing in five patients, leaving 100 patients with blood samples at Day 1, and two patients had no urinary sample. A total of 265 blood samples and 277 urinary samples were analyzed.

**Table 1:** Baseline characteristics of the 105 patients

	Mean ± SD	Nr (%) or Median (IQR)
Age (ys)*	65.24 ± 14.57	66 (55.5 to 75.0)
Male sex		70 (66.7)
Body weight (kgs)*	81.1 ± 17.3	80 (70 to 90)
BMI	27.2 ± 5.6	26.3 (23.5 to 29.3)
Admission type		
• Medical		79 (75.2)
• Cardiosurgical		10 (9.5)
• Other surgical		16 (15.2)
Sepsis		34 (32.4)
CRP admission (mg/L)	89.1 ± 116	26.0 (3.0 to 160.6)
APACHE II score	27.4 ± 9.0	26.5 (20.0 to 34.0)
APACHE II PM	0.57 ± 0.28	0.59 (0.31 to 0.84)
APACHE III score	102 ± 35	97.5 (73.0 to 125.8)
APACHE IV PM	0.55 ± 0.31	0.56 (0.26 to 0.85)
SOFA admission	9 ± 4	9 (7 to 11)
Mechanical ventilation		104 (99.0)
MAP admission (mmHg)	80.6 ± 21.9	79 (62.0 to 94.0)
Dopamine admission (μg/kg/minute)	11.1 ± 13.7	5.9 (2.4 to 14.3)
Noradrenalin admission (μg/kg/minute)	0.05 ± 0.13	0.00 (0.00 to 0.04)
Lowest pH ^a ^	7.2 ± 0.18	7.2 (7.08 to 7.30)
Lowest bicarbonate ^a^	17.8 ± 6.2	17.3 (13.8 to 22.3)
Lowest PO_2 _(mmHg) ^a^	74.0 ± 23.0	70 (60.3 to 82.0)
PF ratio (mmHg/fraction)	244 ± 144	206 (151 to 294)
Creatinine before admission^b ^	80 ± 31	77 (56 to 98)
Creatinine (μmol/L)	118 ± 86.6	98.5 (71.0 to 133.3)
Urea (mmol/L)	11 ± 7	8 (6 to 14)
Urinary output d1(ml)	2,711 ± 1,851	2,378 (1,174 to 3,969)
Urinary output d1(ml/kg/h)	1.5 ± 1.1	1.2 (0.6 to 2.1)
RIFLE 1,2,3 ^c^		
0	60 (58.3)	
1	15 (14.6)	
2	16 (15.5)	
3	12 (11.7)	
Chronic dialysis		2 (2%)
Bilirubin (μmol/L)	14 ± 17	9 (5 to 16)
Hemoglobin (mmol/L)	6.8 ± 1.6	6.8 (5.8 to 8.2)
Platelets (giga)	236 ± 144	209 (151 to 298)

* Normally distributed variables.

APACHE, Acute Physiology and Chronic Health Evaluation; IQR, interquartile range; PM, predicted mortality; SAPS, Simplified Acute Physiology Score; SD, standard deviation; SOFA, Sequential organ failure score.

^a ^within two hours of ICU admission, ^b ^creatinine of the chronic dialysis patients not included, ^c ^RIFLE acronym for risk of acute kidney injury: Risk = 1, Injury = 2, Failure = 3. The chronic dialysis patients were excluded from the RIFLE classification.

**Figure 1:**
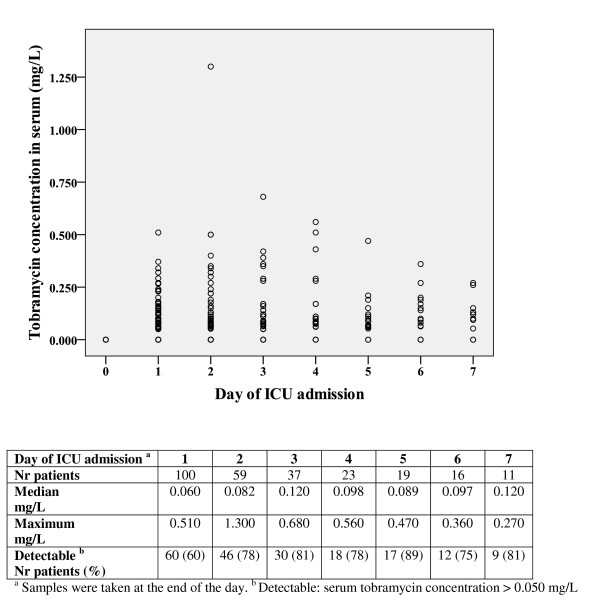
**Serum tobramycin concentrations and percentage of patients with detectable serum tobramycin (> 0.050 mg/L)**. Day 0: on admission. Day 1 to Day 7: serum tobramycin concentration at the end of the day.

Eighty-three out of 100 patients with available blood samples (83%) had once had at least a detectable serum tobramycin concentration (> 0.050 mg/L). Tobramycin concentrations in serum during the first seven days are represented in Figure 1. Serum tobramycin concentration at Days 3, 4 and 5 was significantly higher than at Day 2 (*P *< 0.05), but stabilized thereafter. The number of patients decreased per day due to discharge or death. The median highest serum tobramycin concentration per patient was 0.120 (IQR 0.063 to 0.232) mg/L. A serum concentration > 1 mg/L was detected once in one patient at 48-h after ICU admission (Figure 1).

**Figure 2:**
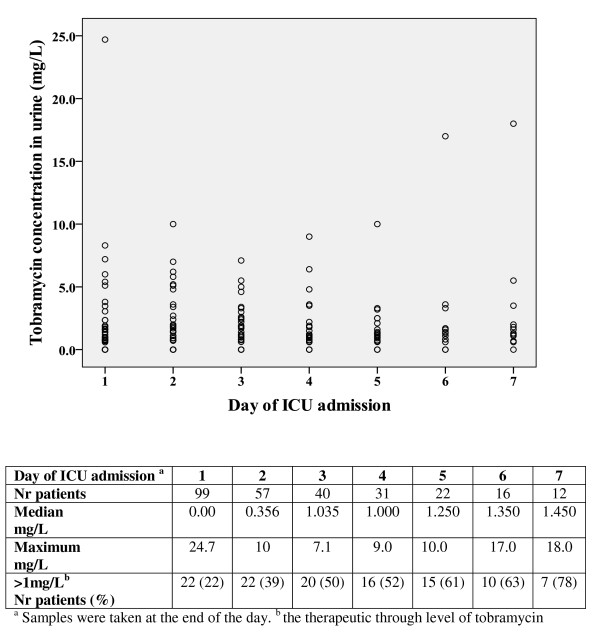
**Tobramycin concentrations in 24-h urine and percentage of patients with a urinary tobramycin concentration > 1.0 mg/L)**.

A total of 102 patients (99%) had at least one urinary sample with detectable tobramycin (> 0.50 mg/L), while 50 patients (49%) had at least one urinary sample with a tobramycin concentration ≥ 1 mg/L. The tobramycin concentration in urine increased by day of admission up to Day 5 and remained fairly constant thereafter (Figure 2). The proportion of patients with a urinary tobramycin concentration > 1 mg/L increased by admission day, and was more than half from Day 4. The median total amount of tobramycin recovered in urine per day varied between 2 to 4.2 mg/day (about 1% of the daily dose) and was 25 mg/day at most (8% of the daily dose).

We correlated highest serum tobramycin concentration with markers of organ failure and inflammation on the same day and on those on the day of ICU admission. Dopamine, our first line vasopressor at that time, was used in 94 patients, while 32 patients received additional noradrenalin. Only the strongest correlations are reported. Highest tobramycin concentration correlated positively with C-reactive protein (CRP) (r = 0.26, *P *= 0.01), urea (r = 0.21, *P *= 0.047) and bilirubin (0.30, *P *= 0.005) on admission, to highest dopamine dose (r = 0.21, *P *= 0.04), noradrenalin dose (r = 0.20, *P *= 0.05) and urinary output (ml/kg/h) on Day 1 (r = -0.25, *P *= 0.01), and to RIFLE based on creatinine during the study period (r = 0.22, *P *= 0.03), and correlated negatively with gastric retention on Day 1 (r = -0.24, *P *= 0.023). Highest tobramycin concentration was significantly higher in patients without AKI compared to those with any degree of AKI according to RIFLE, (median 97 (IQR 51 to 172) versus 130 (IQR 84 to 285), *P *= 0.04). Highest serum tobramycin concentration was not significantly related to total SOFA and APACHE scores.

We subsequently compared the clinical characteristics of the patients with tobramycin leakage to those without. It appeared that differences in markers of organ failure were most pronounced at the day of ICU admission. These are presented in Table 2. Patients with tobramycin leakage received more dopamine and had a lower diuresis at the day of ICU admission; they tended to be older, to receive more noradrenalin, to have a higher bilirubin and tended to have a higher CRP. In subsequent binary logistic regression analysis, dopamine dose (odds ratio 1.13 (95% CI 1.01 to 1.34) and urinary output on Day 1 (odds ratio 0.54 (95% CI 0.33 to 0.90) remained as significant predictors of tobramycin leakage. Tobramycin leakage was associated with high dopamine dose and low urinary output. ICU length of stay and gastric retention did not confound the model.

**Table 2:** Comparison of clinical characteristics at Day-1 and outcome between patients with tobramycin leakage and without

	Without tobramycin leakage *n *= 17	With tobramycin leakage *n *= 83	*P*-value
**Baseline**			
Age (years)	62 (49.5 to 69.5)	68 (59.0 to 76.0)	0.06
BMI	25.2 (22.7 to 27.7)	27.2 (24.1 to 29.3)	0.11
SOFA score	10 (7 to 11)	9 (7 to 12)	0.60
APACHE II score	25 (21 to 33)	27 (20 to 35)	0.61
APACHE II PM	59 (40 to 83)	61 (31 to 84)	0.78
SAPS II score	58 (46 to 66)	59 (44 to 73)	0.81
SAPS II PM	64 (37 to 78)	65 ((33 to 87)	0.79
APACHE III score	101 (70 to 124)	97 (73 to 132)	0.96
APACHE IV PM	61 (25 to 82)	56 (25 to 90)	0.87
Sepsis	5 (29.4)	27 (32.5)	1.00
CRP (mg/L)	3 (2 to 94)	37 (4 to 169)	0.06
Lowest MAP (mmHg)	78 (65 to 99)	79 (61 to 94)	0.90
Dopamine dose (μg/kg/minute)	3.3 (2.2 to 5.9)	6.7 (2.8 to 17.2)	0.02
Noradrenalin dose (μg/kg/minute)	0.00 (0.00 to 0.00)	0.00 (0.00 to 0.05)	0.09
Fluid balance (ml)	3,450 (548 to 4,646)	3,074 (1,323 to 4,528)	0.99
Lowest PO_2 _mmHg	75 (61.0 to 83.5)	70 (60.8 to 82.0)	0.53
PF ratio (mmHg/fraction)	206 (125 to 276)	206 (156 to 300)	0.72
Lowest pH	7.21 (7.08 to 7.27)	7.21 (7.09 to 7.29)	0.68
Creatinine before hospital admission (μmol/L)	83 (64.0 to 101.5)	77 (56.0 to 96.5)	0.43
Creatinine admission (μmol/L)	98 (75 to 129)	99 (69 to 144)	0.92
Creatinine d2 (μmol/L)	87 (59 to 101)	95 (70 to 144)	0.24
Urinary output (ml)	3,645 (1,893 to 4,608)	2,150 (1,092 to 3,585)	0.05
Urinary output (ml/kg/h)	1.8 (1.07 to 2.85)	1.0 (0.55 to 1.88)	0.04
RIFLE 1,2,3 ^c^			
0	13 (77)	45 (56)	0.40
1	2 (12)	11 (14)	
2	1 (6)	14 (17)	
3	1 (6)	11 (14)	
Bilirubin (μmol/L)	6 (4 to 13)	9 (6 to 17)	0.07
Hemoglobin (mmol/L)	7.5 (5.5 to 8.8)	6.8 (5.9 to 8.2)	0.34
Platelets (giga)	214 (165 to 276)	200 (148 to 300)	0.75
Gastric retention (ml/day)	450 (215.0 to 777.0)	250 (112.5 to 660.0)	0.18
**Outcome**			
CVVH during ICU admission	3 (18%)	22 (27%)	0.55
Duration mechanical ventilation (h)	42 (32 to 76)	62 (25 to 114)	0.72
LOS ICU (days)	2.2 (1.8 to 4.0)	3.4 (2.0 to 6.2)	0.29
Creatinine _hospital discharge _(μmol/L)	99 (59 to 129)	82 (58 to 147)	0.69
Creatinine _hospital discharge_**/**creatinine _pre-admission_	1.13 (1.02 to 1.32)	1.16 (1.00 to 1.54)	0.81
Mortality hospital n (%)	6 (35)	25 (30)	0.46

Values in median (interquartile range). APACHE, Acute Physiology and Chronic Health Evaluation; CVVH, continuous venovenous hemofiltration; PF, ratio PaO2 (mmHg)/inspiratory oxygen (fraction); PM, predicted mortality; SAPS, Simplified Acute Physiology Score; SOFA, Sequential organ failure score. ^a ^within two hours of ICU admission, ^b ^creatinine of the chronic dialysis patients not included, ^c ^RIFLE acronym for risk of acute kidney injury: Risk = 1, Injury = 2, Failure = 3

To estimate possible renal toxicity of the leaked tobramycin, we compared the serum creatinine _at discharge _with creatinine _pre hospital admission_, and the change in creatinine, expressed as the creatinine _at discharge _divided by creatinine _pre ICU admission_, excluding the three chronic hemodialysis patients. Creatinine pre-admission could be retrieved from 100/105 patients. We found that the two were not significantly different between groups (Table 2). The change in creatinine did not significantly correlate with highest serum tobramycin concentration (*P *= 0.29). None of the patients with AKI remained dialysis-dependent after hospital discharge.

## Discussion

The present prospective cohort study in critically ill patients acutely admitted to the ICU receiving enteral tobramycin as a component of SDD showed that traces of tobramycin could be detected in the serum of 83% of the patients, indicating gut barrier failure. Tobramycin leakage was associated with the severity of shock and inflammation at ICU admission and subsequent AKI; however, nephrotoxicity could not be detected. The study further shows that tobramycin was detectable in urine in 99% of the patients, and that the urinary concentration was above the therapeutic trough level (> 1 mg/L) in half of the patients.

### Clinical determinants of tobramycin leakage

SDD is used in critically ill patients because it effectively reduces colonization of the oropharyngeal and intestinal mucosa and subsequent infections with Gram-negatives and yeasts [1,2]. It is generally assumed that the enterally administered antibiotics remain in the digestive tract and are not absorbed. This was the case in 17% of the critically ill patients in this study. In contrast, the present study shows that in the majority of acute critically ill patients receiving SDD, trace amounts of the enteral tobramycin leak into the bloodstream. Tobramycin leakage was best explained by the degree of shock at ICU admission, as roughly reflected by dopamine dose and urinary output at the day of admission, but less so at subsequent days. Highest tobramycin concentration was associated with markers of circulatory dysfunction, liver dysfunction and inflammation on the day of ICU admission, and to acute renal impairment during the study period as reflected by the RIFLE class. Tobramycin leakage was not associated with total SOFA score, which also includes neurological dysfunction. Unfortunately, we did not routinely measure lactate at the time of the study and could, therefore, not correlate this marker of poor circulation with highest tobramycin. These results suggest that gut barrier failure was likely due to temporary gut ischemia due to shock and systemic inflammation, and that the absorbed tobramycin slightly accumulated until Day 5 due to AKI. Although it may be hypothesized that small amounts of tobramycin always leak into the circulation and that blood concentrations only rise in patients with some kidney injury, this is likely not the case, because in 17% of the patients tobramycin was never detected in the blood despite using an extremely sensitive detection method. Possible sequels of tobramycin leakage will be discussed.

### Risk of toxicity

Tobramycin toxicity is associated with high prolonged serum concentrations [14,15] and especially high trough concentrations [16]. Persistent leakage of tobramycin may be toxic, especially for the inner ear and kidneys [5,6,17]. Tobramycin could be detected in the serum in 83% of the patients when using a sensitive method (detection > 0.050 mg/L). Previous studies using a slightly less sensitive method (detection > 0.18 mg/L) found detectible serum tobramycin concentrations in 9/15 ventilated patients (> 1 mg/L in 2 patients) [18], in 56% of the samples from 22 intensive care patients receiving SDD for 10 days with concentrations > 2 mg in the 2 patients with renal failure [19], and in 12/19 critically ill patients treated with continuous venovenous hemofiltration (> 1 mg in 4 patients) [20]. We found a concentration > 1 mg/L only once and a steady state after repeated administrations (see Figure 1). Recently, a serum tobramycin concentration of 18.9 mg/L was reported 30 days after oesophago-gastrectomy. The patient developed AKI and remained dialysis dependent. Toxic tobramycin concentrations were likely explained by colonic ischemia, motility disorders, a blind jejunal loop and additional suppositories [21]. Mild toxic effects are difficult to determine in critically ill patients without a randomized controlled design due to the complexity of critical illness and its associated multiple drug use and also because tobramycin leakage during the first ICU day was already associated with oliguria at ICU admission, before any tobramycin was administered. We could not show major kidney injury due to tobramycin leakage, because creatinine at hospital discharge and change in creatinine (at discharge divided by pre-hospital admission) were not higher in the patients with tobramycin leakage. We did not take audiograms in our patients, thus otoxicity was not monitored. Fortunately, tobramycin leakage in our patients was not associated with crude outcome markers like ICU stay or hospital mortality. To prevent possible toxicity of SDD, monitoring blood tobramycin concentrations should be considered in patients with a prolonged vasopressor-dependent circulation, renal dysfunction, enteral dysmotility and especially when adding tobramycin suppositories or nebulization in patients. When serum concentrations remain > 1 mg/L, enteral tobramycin dose should be reduced.

### Antimicrobial effects

Although trace concentrations of tobramycin could be detected in the blood in the majority of the patients, blood concentrations were above the therapeutic trough concentration only once and far from sufficient to treat blood stream infections. In contrast, urinary tobramycin concentrations unexpectedly were above the therapeutic trough concentration in half of the patients from Day 3 and in two-thirds at the end of the first week of ICU admission. Urinary concentrations up to 24 mg/L were measured and concentrations increased until Day 5. Tobramycin is entirely removed from the body by glomerular filtration, 85% within 24 hours when renal function is normal [4,15,22]. The remaining drug accumulates, particularly in renal cortical tissue, and is slowly excreted by urine for 10 to 20 days [23,24], explaining the increasing proportion of patients with urinary concentrations > 1 mg/L in time. Urinary tobramycin concentration depends on plasma concentration, on glomerular filtration rate with a delay, and on tubular concentration ability. Urinary concentrations are thus manifold higher than plasma concentrations, even with renal dysfunction as present in some of our patients. The presence of tobramycin in urine in patients with gut barrier failure, as first shown in this study, may contribute to the prevention of urinary tract colonization with tobramycin sensitive strains. Unfortunately, we cannot support this assumption with data from urinary cultures. In the past, we have discontinued taking urinary cultures as part of the SDD surveillance, because we almost never found Gram negative bacteria in urinary cultures. Elimination of the enteral focus of urinary Gram negatives is one mechanism explaining the absence of urinary Gram negatives; however, direct urinary antimicrobial effects by urinary tobramycin originating from the gut, as demonstrated in the present study, may additionally prevent urinary tract colonization with Gram-negative bacteria in the setting of the low prevalence of multi-resistant strains.

### Risk of resistance

Prolonged exposure of blood and urine to low concentrations of tobramycin might promote tobramycin resistance. However, the fear of tobramycin resistance in the setting of SDD does not seem realistic. Resistance is more likely to occur if a high bacterial load is exposed to sub-therapeutic antibiotic concentrations. The highest bacterial load in the ICU resides in the patient's gut. During SDD gut concentrations are extremely high (320 mg/day), creating unfavorable conditions for the development of resistance. This is supported by a Cochrane meta-analysis concluding that the risk of resistance using SDD was appropriately explored in only one trial, which found that SDD substantially decreased colonization with resistant organisms [1,25]. Recently, the largest study up to now, a multi-center open-label, clustered group-randomized, crossover study, analyzing data from 5,463 patients, who were in the ICU for more than three days, found a decrease in acquired bacteremia and respiratory tract colonization with highly resistant microorganisms during SDD [26]. Although the use of SDD can select resistant strains not susceptible to polymyxin or tobramycin [27], there are no indications that SDD promotes tobramycin resistance.

### Strength and limitations

The present study presents the largest cohort of critically ill patients up to now with serial serum tobramycin measurements during SDD over a longer time frame, and also the largest study that relates tobramycin leakage to markers of organ failure and clinical outcome. It is the first study presenting urinary concentrations and showing that these concentrations are substantial. Furthermore, we used the most sensitive method to detect serum tobramycin. Its limitation is that the analysis of clinical variables associated with tobramycin leakage is difficult, because follow-up time varied between patients due to differences in ICU stay. We, therefore, choose two single markers of tobramycin leakage per patient, a quantitative marker for correlation (the highest tobramycin concentration for each patient), and a qualitative marker for comparing groups with and without tobramycin leakage (at least once a positive serum sample). We did not take into account the area under the curve, because of a different follow-up between patients. An additional difficulty in the interpretation of associations between tobramycin leakage to clinical variables is the lag time between renal dysfunction and the rise of tobramycin. This may explain why correlations at the same day were less strong than those on the day of ICU admission. Patients are generally the sickest at ICU admission, while it takes some days before tobramycin levels increase due to AKI. However, the results are biologically plausible. Unfortunately, ototoxicity cannot be monitored in critically ill patients, while renal toxicity can only be assessed in a large prospective controlled design if baseline renal function is documented. Potential toxicity can, however, not erase the positive effect of SDD on infection prevention and patient outcome as shown in previous studies [1,2].

## Conclusions

The present observational cohort study in acutely admitted critically ill patients receiving SDD shows that trace amounts of the enterally administered tobramycin were detectable in the serum of the majority of the patients during the first week of ICU admission. Tobramycin leakage was associated with the severity of shock and inflammation at ICU admission and subsequent AKI, suggesting gut barrier failure and subsequent decreased removal. Unexpectedly, urinary tobramycin concentrations were above the therapeutic trough level in half of the patients. The presence of tobramycin in urine in critically ill patients with gut barrier failure likely contributes to the prevention of urinary tract colonization with tobramycin sensitive strains. The present findings do not indicate that we should stop treating our patients with SDD. However, they underscore the potential of tobramycin leakage to the systemic circulation and its possible systemic side effects. SDD remains an important infection prevention strategy. To prevent possible toxicity during prolonged use of SDD, we should consider monitoring serum tobramycin concentrations in patients at risk and reducing the enteral dose when serum concentrations remain > 1 mg/L.

## Key messages

• During the use of selective decontamination of the digestive tract (SDD), trace amounts of the enterally administered tobramycin are present in the blood of the majority of critically ill patients acutely admitted to the ICU.

• Tobramycin leakage is associated with the severity of shock and inflammation at ICU admission and subsequent AKI; however, nephrotoxicity could not be shown.

• Tobramycin leakage reflects loss of the gut barrier and subsequent decreased removal.

• To prevent possible toxicity during prolonged use of SDD, monitoring of serum tobramycin concentrations in patients at risk should be considered, and the enteral tobramycin dose reduced when serum concentrations remain > 1 mg/L.

• Urinary tobramycin concentrations were above the therapeutic trough concentration in half of the patients during the first week of ICU admission and may, therefore, contribute to the prevention of urinary tract colonization with tobramycin sensitive strains.

## Abbreviations

AKI: acute kidney injury; APACHE: Acute Physiology and Chronic Health Evaluation; CI: confidence interval; CRP: c-reactive protein; IQR: interquartile range; OR: odds ratio; RIFLE: Risk, Injury, Failure, Loss, End Stage Kidney disease; SD: standard deviation; SDD: selective decontamination of the digestive tract; SOFA: Sequential Organ Failure Assessment.

## Competing interests

The authors declare that they have no competing interests.

## Authors' contributions

HMOvS initiated the study, participated in the design, coordinated the implementation of the study, participated in the statistical analysis and was the primary writer of the manuscript. HE participated in the statistical analysis and the drafting and writing of the manuscript. RJB collected and extracted the clinical data and participated in the writing. MEAdJ participated in the development of the tobramycin assay and carried out the tobramycin measurements. MlvO contributed to the microbiological interpretation of the findings and the writing of the manuscript. DFZ participated in the interpretation of the results in the context of the literature. EJFF initiated the development of the tobramycin assay, was responsible for the final tobramycin results and contributed to the writing process. All authors read and approved the final manuscript for publication.
